# Exosomes Derived From Mesenchymal Stem Cells Regulating Myotube Cell Atrophy via NF‐κB Signaling Pathway

**DOI:** 10.1155/sci/3606738

**Published:** 2026-04-22

**Authors:** Yibing Ke, Yongping Wang

**Affiliations:** ^1^ Beijing Friendship Hospital, Capital Medical University, Beijing, China, ccmu.edu.cn; ^2^ Department of Orthopedics, First Hospital of Lanzhou University, No.1 Donggang West Road Chengguan, Lanzhou, China, lzu.edu.cn; ^3^ Department of Orthopaedics, The Second Affiliated Hospital of Hainan Medical University, Haikou, China, hainmc.edu.cn

**Keywords:** bone mesenchymal stem cells (BMSCs), C2C12, exosomes, myotube atrophy, NF-κB signaling pathway, tumor necrosis factor‑α (TNF-α)

## Abstract

Bone marrow mesenchymal stem cells (BMSCs), with their potential for multidifferentiation and self‐replication, are considered effective for repairing damaged tissues. BMSCs can repair and replace damaged tissues by differentiating into effective cells and secreting some cytokines that inhibit inflammation and promote tissue repair. Recent studies suggest that BMSCs’ main role in tissue repair may involve the secretion of exosomes (BMSC‐Exos). Basic research has shown that exosomes can have significant effects on orthopedic diseases such as osteoarthritis, muscle tissue injury, and fractures by stimulating regeneration and reducing inflammation. However, the effect of exosomes on tumor necrosis factor (TNF‐α)‐induced muscle atrophy remains unclear.

Therefore, this study investigated the mechanism underlying the protective effect of BMSC‐Exos on TNF‐α‐induced C2C12 myotube atrophy. Treatment with TNF‐α (20 ng/mL) for 48 h significantly reduced myotube viability and diameter, which were subsequently reversed by treatment with BMSC‐Exos. BMSC‐Exos treatment suppressed the expression of E3 ubiquitin ligases, including Atrogin‑1/muscle atrophy F‑box and muscle ring‑finger protein‑1 (MuRF‐1). Furthermore, it increased the protein expression levels of myoblast determination protein‐1 (MyoD) in TNF‐α‐induced myotubes. BMSC‐Exos also decreased the nuclear translocation of nuclear factor‐κB (NF‐κB) by inhibiting the phosphorylation of κB inhibitors.

These findings indicate that BMSC‐Exos may protect against TNF‐α‐induced myotube atrophy by inhibiting the proinflammatory NF‐κB pathway.

## 1. Introduction

Skeletal muscle is a significant tissue in the human body, constituting ~40% of total body weight and 50% of total protein. It plays a crucial role in regulating body temperature, whole‐body metabolism, exercise, and visceral protection. Muscle atrophy, which can result from various pathophysiological conditions such as hunger, cancer, diabetes, long‐term bed rest, denervation, or motor neuron disease, is also a common issue in aging individuals. The histological features of skeletal muscle atrophy include reduced muscle mass and fiber cross‐sectional area, weakened contraction, and external manifestations such as diminished strength, decreased athletic ability, and decreased quality of life for patients. Research indicates that muscle atrophy is mainly caused by the overactivation of the ubiquitin (Ub) proteasome system, a major protein degradation pathway within muscle cells. In this system, protein substrates are first labeled with Ub molecules and subsequently hydrolyzed into small peptides by the 26S proteasome complex. In this protein degradation pathway, three types of ubiquitin‐conjugating enzymes (E1, E2, and E3) play major roles in linking Ub to protein substrates [1]. The rate‐limiting step of the ubiquitination process affects subsequent proteasome‐dependent intracellular protein degradation, which is catalyzed by the E3 enzyme. Currently, several E3 ligases upregulated during muscle loss have been identified. These include Atrogin‐1/MAFbx (muscle atrophy F‐box) and muscle ring finger protein‐1 (MuRF‐1) [2]. These proteins are currently recognized as two muscle‐specific protein hydrolysis ubiquitin ligases that promote protein degradation in muscle cells and are considered biomarkers of skeletal muscle atrophy. Their elevated levels are associated with various skeletal muscle atrophy diseases [3, 4].

Atrophy‐related genes upregulated during muscle atrophy transcribe Atrogin‐1 and MuRF‐1 proteins, which are related to the ubiquitin‐proteasome and promote protein degradation. The expression of myogenic differentiation antigen (MyoD) increases during the growth and differentiation of myotubes, thus promoting protein synthesis in myotubes [5]. Atrogin‐1 facilitates the degradation of MyoD and eIF3‐f, a significant protein synthesis activator [6], whereas MuRF‐1 interacts with crucial muscle structural proteins, including troponin 1, myosin heavy chain, actin, and myosin binding protein C, which regulates their half‐lives [7]. Muscle satellite cells, located beneath the basement membrane, are skeletal muscle stem cells that provide muscle nuclei for postnatal muscle growth, repair, and regeneration in adults. Satellite cells are activated during muscle injury, after which they proliferate widely and differentiate into muscle cells. C2C12 cells are mouse myoblast cell lines derived from satellite cells [8]. These cells are commonly used as an in vitro model for muscle regeneration because they can transition from the proliferation phase to differentiate muscle fibers under appropriate stimulation [9].

Proinflammatory cytokines such as tumor necrosis factor‐α (TNF‐α), interleukin (IL)‐1β, and IL‐6 have been shown to promote the breakdown of myofibrillar proteins and reduce protein synthesis, which directly leads to muscle loss [10]. TNF‐α is a proinflammatory cytokine associated with muscle breakdown metabolism and plays crucial roles in various biological processes, including cell growth, differentiation, inflammation, apoptosis, and necrosis. Among its various effects on skeletal muscle proteins, TNF‐α acts as an effective activator of the NF‐κB pathway by activating several intracellular factors, including nuclear factor kappa B (NF‐κB), Atrogin‐1/muscle atrophy F‐box (MAFbx), and muscle ring finger protein‐1 (MuRF‐1). It also induces apoptosis and ubiquitin‐dependent protein hydrolysis [11]. The phosphorylation and proteasomal degradation of the NF‐κB inhibitory protein (IκB inhibitor) lead to the activation and nuclear translocation of NF‐κB [12]. First, NF‐κB increases the protein expression of components of the ubiquitin‐proteasome system, which is involved in the degradation of specific muscle proteins during muscle atrophy [13]. Second, NF‐κB enhances the expression of inflammation‐related factors such as IL‐1β and IL‐6, which directly or indirectly activate muscle atrophy. Third, NF‐κB inhibits the expression of genes related to muscle differentiation, such as myoblast determinant protein‐1 (MyoD), which is involved in the regeneration of atrophied skeletal muscle [14]. MyoD is a myogenic transcription factor crucial for muscle differentiation, and NF‐κB activation decreases MyoD levels through posttranscriptional regulation. Overall, NF‐κB increases the activity of the ubiquitin‐proteasome pathway, promotes muscle protein decomposition, and inhibits muscle production, leading to muscle atrophy.

Bone marrow mesenchymal stem cells (BMSCs), with their potential for multidirectional differentiation and self‐replication, are considered an effective approach to tissue repair [15]. The biological mechanisms induced by BMSCs are multifaceted. BMSCs can repair and replace damaged tissues by differentiating into effector cells [16], while also playing crucial roles involving paracrine factors, such as cytokines, growth factors, immune‐regulatory proteins, and extracellular vesicles (EV, exosomes) [17]. Recently, studies have shown that the role of BMSCs in repairing tissue damage may involve secreted exosomes (BMSC‐Exos). Exosomes are nanosized vesicles with a diameter of 30–200 nm that are secreted by cells and play an important role in intercellular communication [18]. They comprise a bilayer membrane and carry many molecules, including proteins, lipids, and RNA, on their surface or lumen, as well as small noncoding microRNAs (miRNAs) involved in tissue regeneration, such as antiapoptotic and antioxidant miRNAs [19]. Exosome marker proteins include a family of four transmembrane proteins, such as CD9, CD63, and CD81, which are commonly used as identification proteins for exosomes [20]. Exomes also transport various proteins, coding and noncoding genes, and bioactive substances. They participate in intercellular information transmission and regulate cell proliferation, migration, angiogenesis, and phenotype transformation. Owing to their small size, low immunogenicity, and no need for additional culture expansion or delivery procedures [21], EVs offer a cell‐free treatment option that can effectively stimulate cell regeneration while avoiding risks of immune rejection and cell malignancy. This makes them promising candidates for future clinical applications. GW4869, a neutral sphingomyelinase inhibitor, is often used to block exosome generation [22]. It inhibits the release of exosomes from multivesicular bodies (MVBs) by preventing MVB inward budding [23].

Previous studies have shown that EVs derived from BMSCs accelerate the recovery of muscle contraction function in animal muscle strain models [18], whereas EVs isolated from human adipose tissue‐derived BMSCs increase MyoD protein expression in quadriceps injury models [24]. EVs derived from BMSCs also accumulate at the site of spinal cord injury (SCI) and bind with microglia to inhibit the activation of NF‐κB in SCI, thereby promoting recovery in a rat model [25]. However, the efficacy of BMSC‐derived exosomes in preventing muscle atrophy caused by chronic low‐grade inflammation has not been confirmed. Therefore, this study investigated the protective mechanism and therapeutic potential of BMSC‐derived exosomes against TNF‐α‐induced C2C12 myotube atrophy.

## 2. Materials and Methods

### 2.1. The Isolation and Identification of BMSCs

Five clean‐grade, healthy male C57BL/6J mice aged 6–8 weeks (purchased from Lanzhou University Animal Medicine Experimental Center) were euthanized via the cervical dislocation method and soaked in 75% alcohol for 10 min. The femurs and tibias were subsequently harvested under sterile conditions. The bone marrow cavity was rinsed repeatedly with complete DMEM (Gibco, 6124031) containing 10% FBS (Pricella, 164210) and 1% streptomycin mixture (SEVEN, SC118‐01) until the bone marrow cavity turned white. The contents of the bone marrow cavity were collected and centrifuged at 800 × *g* for 3 min. After discarding the supernatant, the cells were suspended in a culture medium and inoculated into T25 culture flasks. The cells were cultivated at 37°C in a 5% CO_2_ incubator. The culture medium was changed daily to remove nonadherent cells. Once the primary cells formed typical proliferative colonies (5–6 days), the cells were passaged. After each passage, nonadherent cells were removed. The surface markers of the isolated BMSCs, including CD29 (BioLegend, 102219) and CD45 (BioLegend, 157213), were detected via flow cytometry.

### 2.2. Isolation and Identification of BMSC‐Derived Exosomes

Third‐generation BMSCs were cultured until they reached 80%–90% confluence and then switched to an FBS‐free medium for 24–48 h. The cell supernatant was collected, and exosomes were isolated using a high‐speed centrifuge (BECKMAN, BECKMAN Optima L‐100XP) to extract the EVs. First, the collected cell supernatant was centrifuged at 4°C and 300 × *g* for 10 min, after which the supernatant was carefully extracted, and the precipitate was discarded. The mixture was subsequently centrifuged at 4°C and 2000 × *g* for 10 min, with the supernatant carefully absorbed and the precipitate discarded. The mixture was centrifuged at 4°C and 10,000 × *g* for 30 min, with the supernatant carefully removed, the precipitate discarded, and the supernatant filtered through a 0.22‐μm filter membrane. The filtrate was then collected and transferred to an ultracentrifugation tube. The mixture was centrifuged at 4°C and 100,000 × *g* for 70 min, the supernatant was discarded, and the precipitate was resuspended in PBS The mixture was incubated at 4°C and 100,000 × *g*, and centrifuged again for 70 min. After discarding the supernatant, the obtained precipitate was resuspended in 150 μL of PBS buffer and the mixture was stored at −80°C for later use.

1. BMSC‐Exos samples obtained from the above experiments were dropped onto a transmission electron microscope copper mesh, 3% tungsten phosphate was added dropwise, and the mixture was allowed to stand at room temperature for 2 min. The mesh was cleaned with distilled water, the structure of the exosomes was observed via transmission electron microscopy (Hitachi, HT7800), and images were captured.
2. The BMSC‐Exos samples were dissolved in PBS (filtered through a 0.22‐µm filter membrane) to a final volume of 1 mL. The particle size of the EVs was measured via a zeta potential particle size analyzer (Malvern Panalvirtual Ltd., Zetasizer Pro).

The extracted BMSC‐Exos mixture was mixed with an equal volume of protein lysis buffer, and lysis was performed in an ice bath. To fully lyse the mixture, it was shaken every 5 min for 50 min. The protein mixture via lysing was centrifuged at 4°C and 12,000 rpm for 15 min at 4°C, and the precipitate was discarded. The protein concentration of the supernatant was measured, and a 5× protein denaturation buffer was added. The mixture was boiled at 100°C for 5 min, and the denatured protein sample was stored at −20°C for later use. SDS‐PAGE was used to separate proteins for 80 min. The proteins were transferred to a PVDF membrane and blocked with 5% skim milk at room temperature for 90 min. The membranes were incubated overnight at 4°C with primary antibodies (CD9, Affinity, AF5139; CD63, Affinity, AF5117; CD81, Proteintech, 27855‐1‐AP; Calnexin, Proteintech, 10427‐2‐A). The membrane was washed with TBST three times for 10 min each. After washing, secondary antibodies (HRP, goat antirabbit IgG, biopm, PMK‐014‐090; HRP, goat antimouse IgG, biopm, PMK‐014‐091) were added, and the samples were incubated at room temperature for 1 h. The film was subsequently washed again with TBST three times for 10 min each, after which an enhanced chemiluminescence (ECL) solution was added for development with a high‐sensitivity multifunctional imaging instrument.

### 2.3. C2C12 Cell Culture and Differentiation

At first, to validate the specificity of GW4869 in suppressing BMSC‐Exos secretion, we quantified the exosome protein concentration from Control BMSCs and GW4869‐treated BMSCs. Briefly, BMSCs were divided into two groups: Control BMSCs (cultured without GW4869) and GW4869‐treated BMSCs (cultured with 10 μmol/L GW4869). After 48 h of incubation, exosomes were isolated from the supernatant of both groups using differential ultracentrifugation. The total protein concentration of exosomes, reflecting exosome yield, was determined by BCA assay (Solarbio, PC0020) following the manufacturer’s protocol. The C2C12 myoblasts, derived from mouse skeletal muscle (purchased from Wuhan Ponosi Life Science and Technology Co., Ltd., China), were cultured in DMEM supplemented with 10% fetal bovine serum at 37°C and 5% CO_2_ in a culture incubator. To determine the optimal concentration of BMSC‐Exos for reversing TNF‐α‐induced C2C12 myotube atrophy and ensure experimental reproducibility, we performed a pre‐experiment to optimize the BMSC‐Exos concentration. Briefly, C2C12 cells were divided into five groups: (1) Control group (without TNF‐α and BMSC‐Exos); (2) TNF‐α group (treated with 20 ng/mL TNF‐α); (3) TNF‐α + 20 μg/mL BMSC‐Exos group; (4) TNF‐α + 50 μg/mL BMSC‐Exos group; (5) TNF‐α + 100 μg/mL BMSC‐Exos group. After 48 h of treatment, cell viability was detected by CCK‐8 assay. When the cell confluence reached 80%–90%, the growth medium was replaced with DMEM supplemented with 2% horse serum (Cytiva, SH30074.03) to initiate myotube differentiation(C2C12 cells used for differentiation and subsequent experiments were strictly limited to the passage range of 3–10 to ensure stable myogenic potential). The differentiation medium was changed every 48 h, and the cells were differentiated for 6 days until 90%–100% of them had fused into myotubes. The differentiated C2C12 myotubes were then divided into four groups: the control group (cells cultured in FBS‐free medium); the TNF‐α (Novoprotein, CF09) group (cells treated with 20 ng/mL TNF‐α); the BMSC‐Exos group (cells treated with 20 ng/mL TNF‐α and BMSC‐Exos); and the BMSC‐Exos (GW4869) group (cells treated with 20 ng/mL TNF‐α and BMSC‐Exos isolated from BMSCs treated with 10 μmol GW4869; KKLMED, KM15381). After 48 h of incubation in a serum‐free medium, the protein was extracted from the cells.

### 2.4. Uptake of Exosomes by Myotubes

After C2C12 cells were induced to differentiate into myotubes under the conditions described above, myotubes were incubated with 100 μg/mL PKH26‐labeled exosomes (PKH26; Solarbio, D0030) for 48 h to allow sufficient internalization. Cells were then fixed with 4% paraformaldehyde (Servicebio, G1101‐500 ML) for 15 min, permeabilized with 0.2% Triton X‐100 (Solarbio, T8200) for 10 min, and stained with Phalloidin (Solarbio, CA1620) for cytoskeleton labeling at 37°C for 30 min. Nuclei were counterstained with DAPI (Solarbio, C0065) for 5 min. Finally, the stained cell slides were mounted on glass slides and observed and captured under a confocal laser scanning microscope.

### 2.5. Cell Viability Assay

A 20 µL aliquot of the C2C12 cell suspension was placed on a cell counting plate for counting under a microscope. The cells were inoculated at a density of 10^5^ per well into a 96‐well plate and cultured for 24 h in a constant‐temperature incubator at 37°C and 5% CO_2_. The cells were divided into four groups: TNF‐α, BMSC‐Exos, and BMSC‐Exos (GW4869) groups, with six replicates in each group. After 48 h of treatment with the corresponding drugs, 10 µL of CCK‐8 (Pricella, P‐CA‐001) solution was added to each well of a 96‐well cell culture plate and shaken well. Consequently, the air bubbles were removed from the wells before incubation in a cell culture incubator for 1 h. The cell culture plate was placed into a fully automated enzyme‐linked immunosorbent assay (ELISA) reader (BioTek, SYNERGYH1), the absorbance value (OD value) was measured at a wavelength of 450 nm, and the cell viability was calculated.

### 2.6. Measurement of Myotube Diameter

C2C12 myotube cells were observed and photographed using a phase‐contrast microscope at 100× magnification. Ten random fields were selected from each group, and the middle two‐thirds of each mature myotube were used for diameter measurement. Three random measurements were taken along the length of each of myotube, and the average of these measurements was used to determine the diameter of a single myotube [26, 27].

### 2.7. Western Blot Analysis

Western blotting was used to detect the expression levels of Atrogin‐1 (1∶1000, Bioss, bsm‐54451R), MuRF‐1 (1∶1000, Affinity, DF7187), MyoD (1∶1000, Abmart, TA7733S), GAPDH (1∶50000, Proteintech, 60004‐1‐lg), α‐Tubulin (1∶10000, Proteintech, 66031‐1‐lg), and Lamin B1 (1∶10000, Bioss, bs‐55118R), as well as NF‐κB signaling pathway‐related proteins IκB‐α (1∶1000, ImmunoWay, YT2419), p‐IκB‐α (1∶1000, Abmart, TP56280F), NF‐κB p65 (1∶1000, ImmunoWay, YT3108), and β‐actin (1∶5000, BOSTER, BM3873) in cells. Each group of C2C12 myotube cells was washed twice with precooled PBS at 4°C and collected using a cell scraper. Cytoplasmic proteins were extracted by adding a protease inhibitor‐containing protein extraction reagent (Solarbio, R0050) to the cells, which were then incubated on ice for 10 min. The cells were then centrifuged at 4°C and 12000 × *g* for 10 min, and the supernatant was collected. Nuclear proteins were extracted following the same extraction procedure for cytoplasmic protein. Protein concentrations were measured using the BCA assay (Solarbio, PC0020). The proteins were separated by SDS‒PAGE and transferred to a PVDF membrane. After blocking at room temperature for 1 h, the membrane was incubated with the corresponding primary antibody overnight at 4°C. The following day, the membranes were incubated with the secondary antibody HRP, Goat Anti Rabbit IgG (1∶10000, biopm, PMK‐014‐090), and HRP, Goat Anti Mouse IgG (1∶10000, biopm, PMK‐014‐091) at room temperature for 1 h. ECL luminescent solution was added for development with an ultrasensitive multifunction imaging device, and the expression of target proteins was analyzed.

### 2.8. Statistical Analysis

The results are presented as means ± standard deviations from at least three independent experiments. The data were analyzed using one‐way ANOVA with GraphPad Prism (GraphPad Prism 10.1.2). A *p*‐value of < 0.05 was considered statistically significant.

## 3. Results

### 3.1. Characterization of BMSCs

The morphological characteristics of the BMSCs were observed under an optical microscope. Following the removal of the metaphysis, red blood cells and other cells were extracted from the medullary cavity. After 24 h of culture, red blood cells and tissue debris could be observed, while single BMSCs were present in a star‐like shape at 40× magnification (Figure 1A–D). After 72 h, the BMSCs presented long, spindle‐shaped, and star‐like shapes, clustered into groups (Figure 1E–H). The expression levels of surface markers CD29 and CD45 on the BMSCs were subsequently measured via flow cytometry (Figure 1I,J).

Figure 1Identification results of cell morphology and surface markers. (A–D) The extracted mouse BMSCs were observed under a microscope at different magnifications after 24 h and 72 h. (E–H) The extracted mouse BMSCs were observed under a microscope at different magnifications after 72 h. (I–J) Flow cytometric analysis of the BMSCs surface markers CD29 and CD45 (I: CD29, 99.97%; J: CD45, 0.27%).

**Figure:**
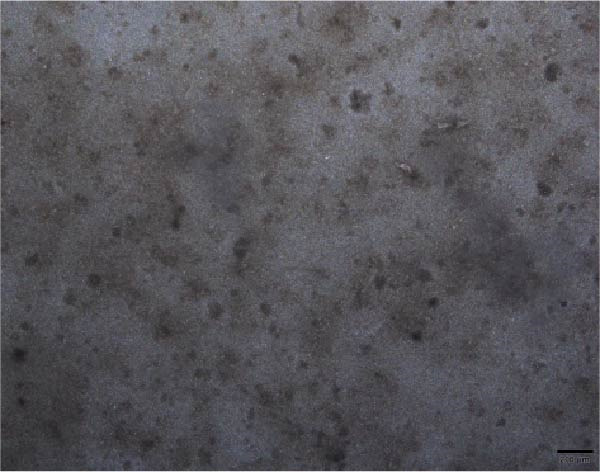
(A)

**Figure:**
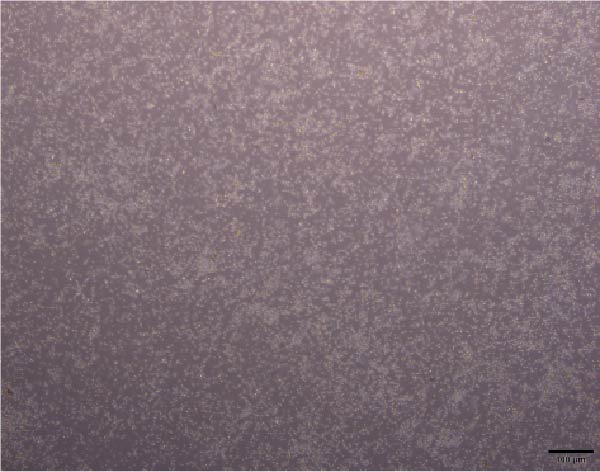
(B)

**Figure:**
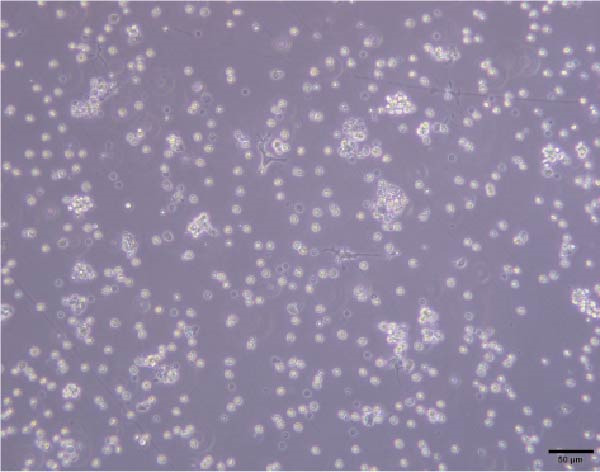
(C)

**Figure:**
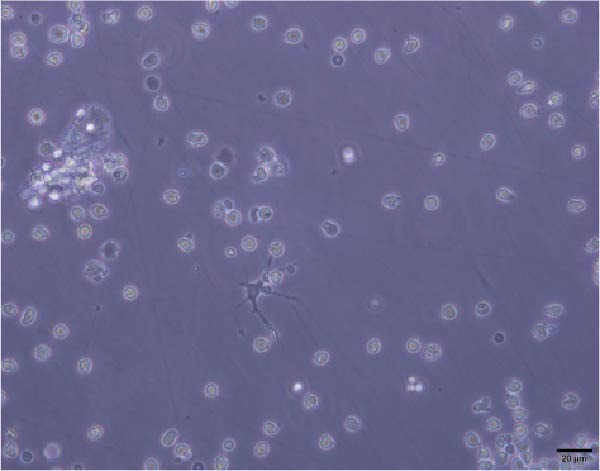
(D)

**Figure:**
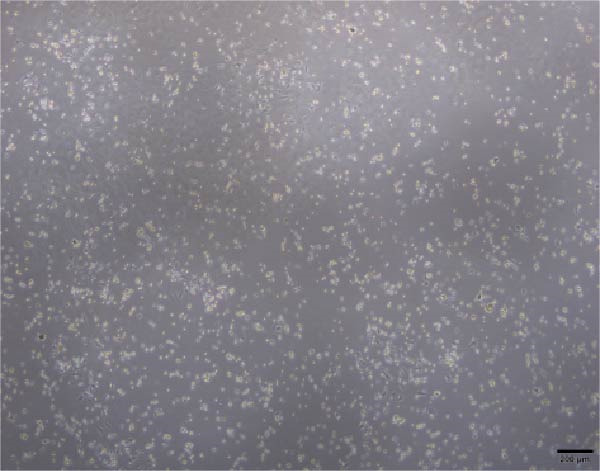
(E)

**Figure:**
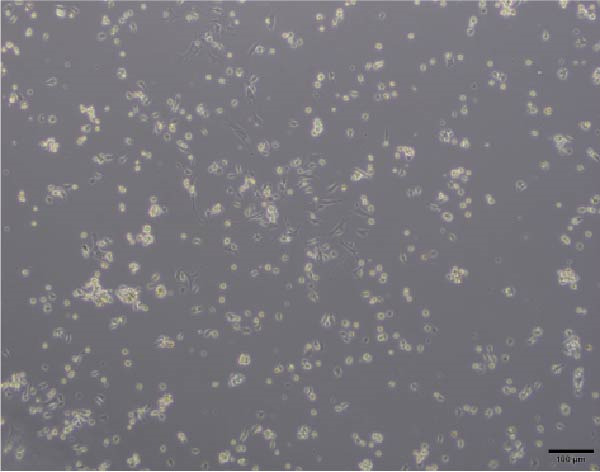
(F)

**Figure:**
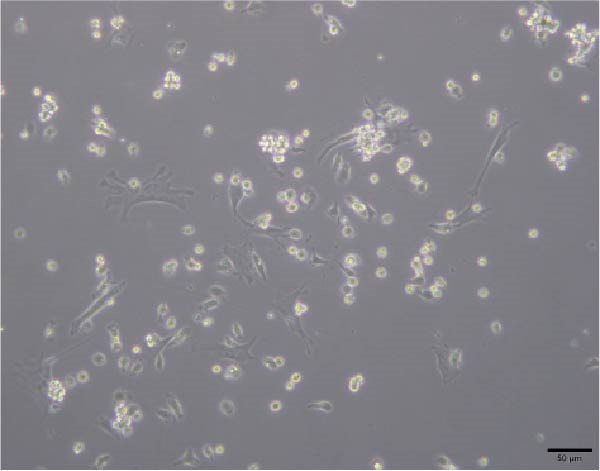
(G)

**Figure:**
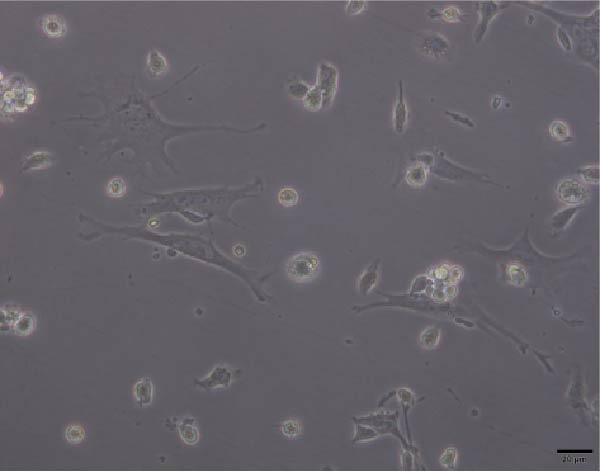
(H)

**Figure:**
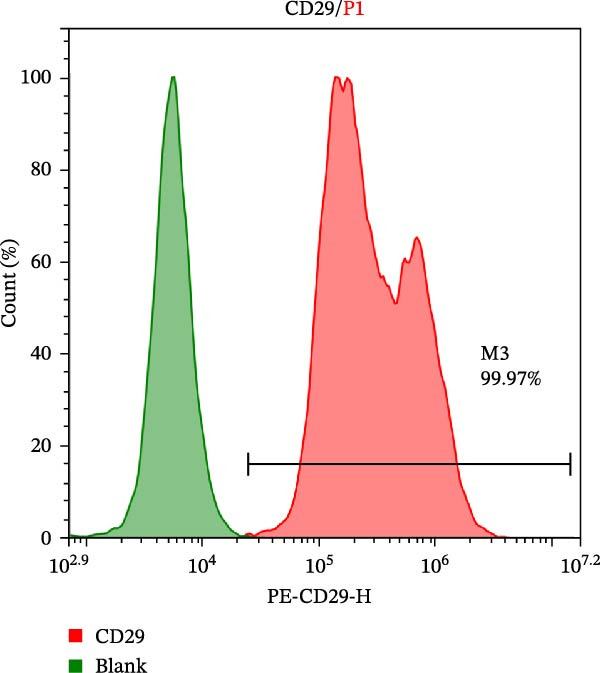
(I)

**Figure:**
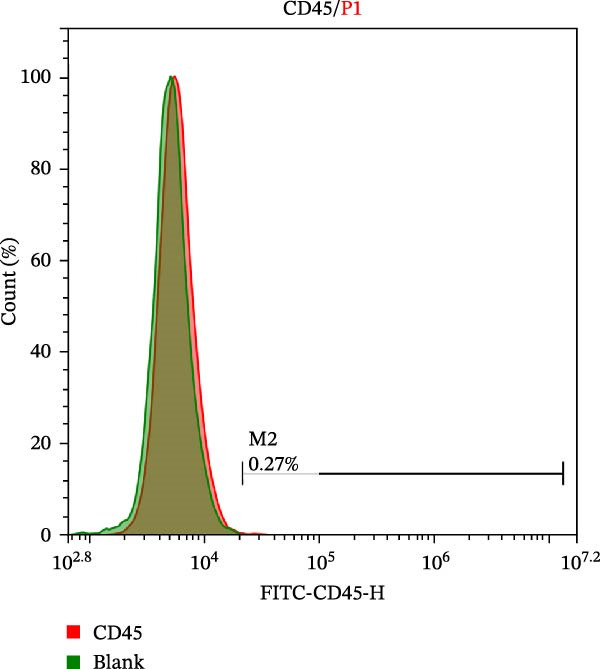
(J)

### 3.2. Characterization of BMSC‐Derived Exosomes

The presence of exosomes was determined by electron microscopy (Figure 2A). The size of the exosomes was assessed using a Zeta potential particle size analyzer, with sizes ranging from 30 to 200 nm (Figure 2B). Nanoparticle tracking analysis (NTA) was performed to determine the particle size distribution and concentration of BMSC‐Exos (Figure 2C). The results revealed a peak particle size at ~100 nm, with a total concentration of 7.9 × 10^9^ particles/mL. These exosomes derived from BMSCs displayed particle size and morphology similar to those described by others. Moreover, Western blot analyses demonstrated that BMSC‐derived exosomes were positive for the specific exosomal markers CD9, CD63, and CD81 (Figure 2D). The results confirmed that our isolation protocol enriched exosome populations from BMSC‐conditioned media.

Figure 2Characterization of BMSC‐derived exosomes. (A) BMSC‐Exos morphology under a transmission electron microscope; (B) Zeta potential particle size analyzer was used to detect the concentration range of the diameter of the BMSC‐Exos; (C) Nanoparticle tracking analysis (NTA) was performed to determine the particle size distribution and concentration of BMSC‐Exos. The results showed a peak particle size at ~100 nm, with a total concentration of 7.9 × 10^9^ particles/mL; (D) Western blot detection of the BMSC‐Exo surface positive marker proteins CD9, CD63, CD81 and negative marker proteins Calnexin.

**Figure:**
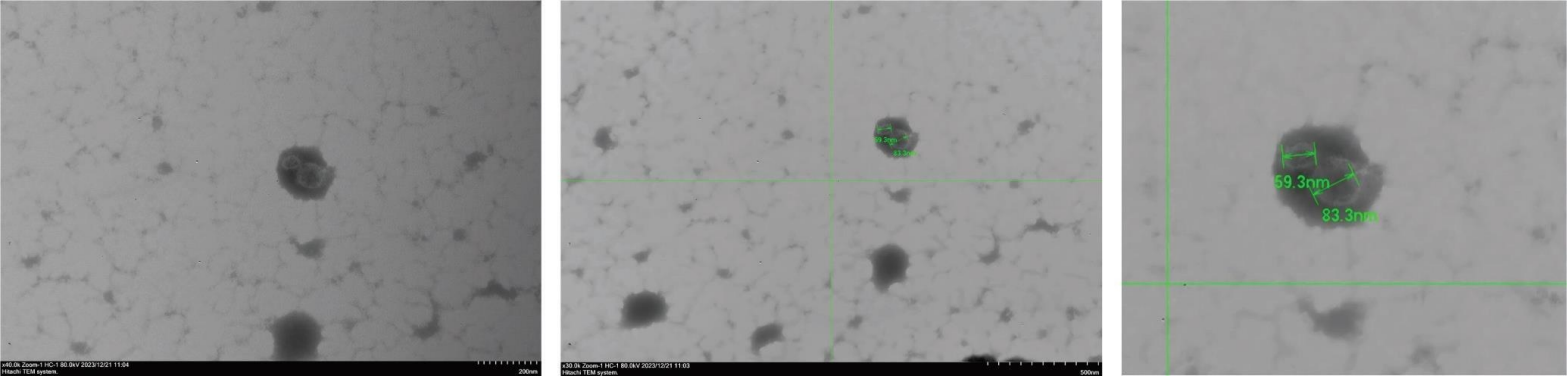
(A)

**Figure:**
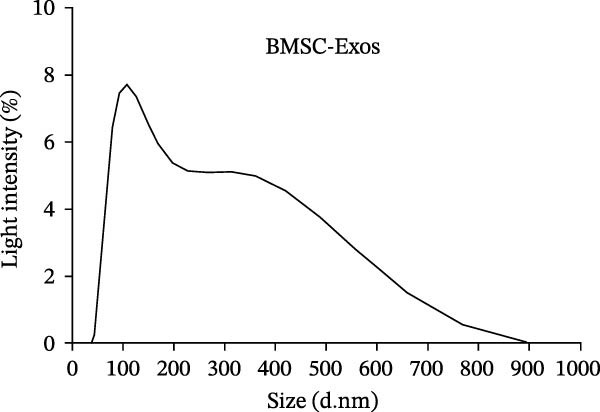
(B)

**Figure:**
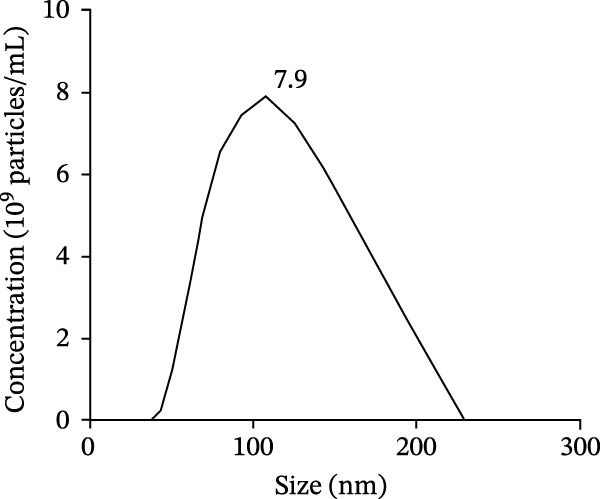
(C)

**Figure:**
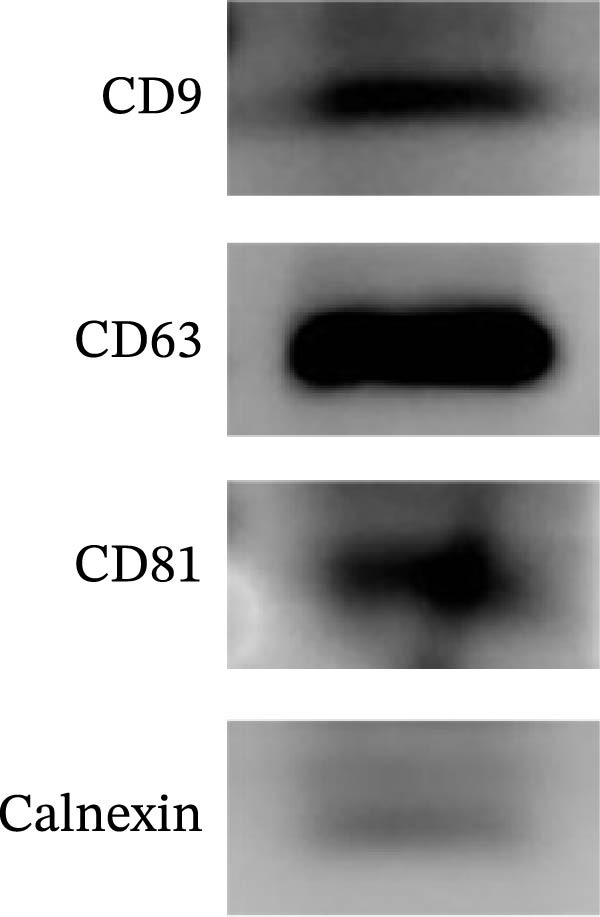
(D)

### 3.3. C2C12 cell Culture and Differentiation

To validate the specificity of GW4869 in suppressing BMSC‐Exos secretion, we measured exosome protein concentration from Control BMSCs and GW4869‐treated BMSCs via BCA assay. The exosome protein concentration was significantly reduced in the GW4869‐treated group compared to the Control group, indicating that 10 μmol/L GW4869 effectively inhibited BMSC‐Exos secretion (Figure 3A). Next, to determine the optimal concentration of BMSC‐Exos that effectively reverses TNF‐α‐induced myotube atrophy, we treated C2C12 cells with different concentrations of BMSC‐Exos (20, 50, or 100 μg/mL) following TNF‐α intervention. The results revealed that BMSC‐Exos exhibited the most potent protective effect when applied at a dose of 100 μg/mL (Figure 3B). Consequently, 100 μg/mL BMSC‐Exos was selected as the working concentration for all subsequent experiments. When C2C12 cells (Figure 3C) reached ~80%–90% confluence, myotube differentiation was initiated by replacing the growth medium with differentiation medium for 6 days, and the medium was changed every 48 h, at which time 90%–100% of the cells had fused into myotubes (Figure 3D). After being differentiated into myotubes, they were divided into a control group, a TNF‐α group, a BMSC‐Exos group, and a BMSC‐Exos (GW4869) group; each group was cultured for 48 h (Figure 4A–D).

Figure 3(A) Exosome protein concentration in Control BMSCs and GW4869‐treated BMSCs. (B) Cell viability of C2C12 cells treated with different concentrations of BMSC‐Exos under TNF‐α stimulation. (C) Morphology of C2C12 cells under a microscope at 200×. (D) Morphology of C2C12 myotubes under a microscope at 200×.  ^∗^ *p* < 0.05,  ^∗∗^ *p* < 0.01,; ^#^ *p* < 0.05, ^##^ *p* < 0.01.

**Figure:**
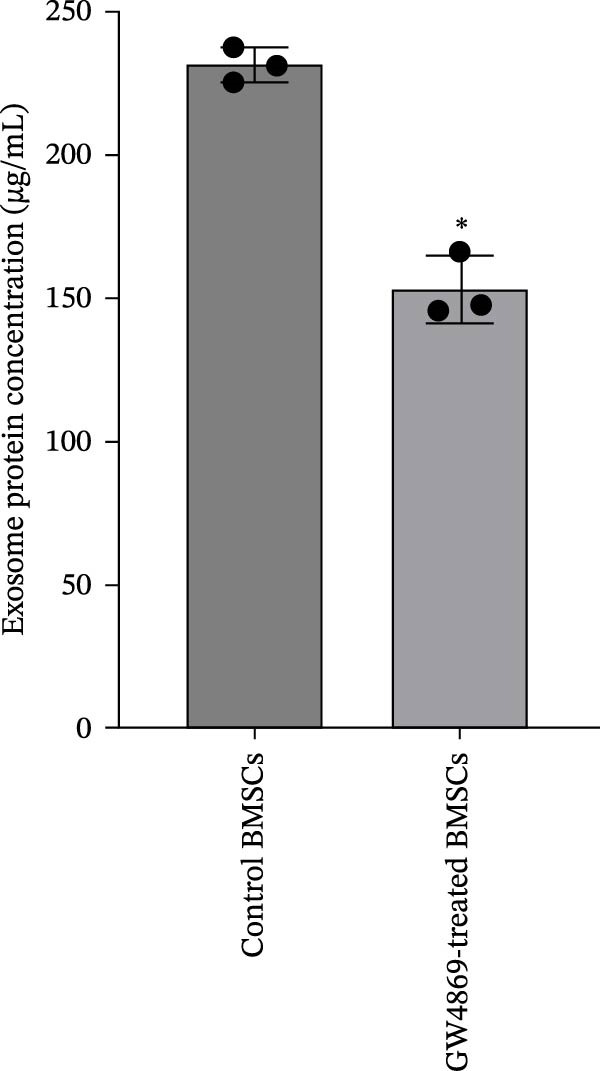
(A)

**Figure:**
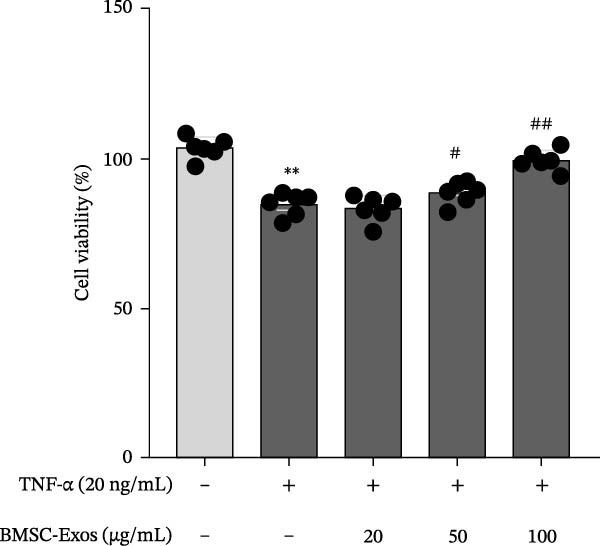
(B)

**Figure:**
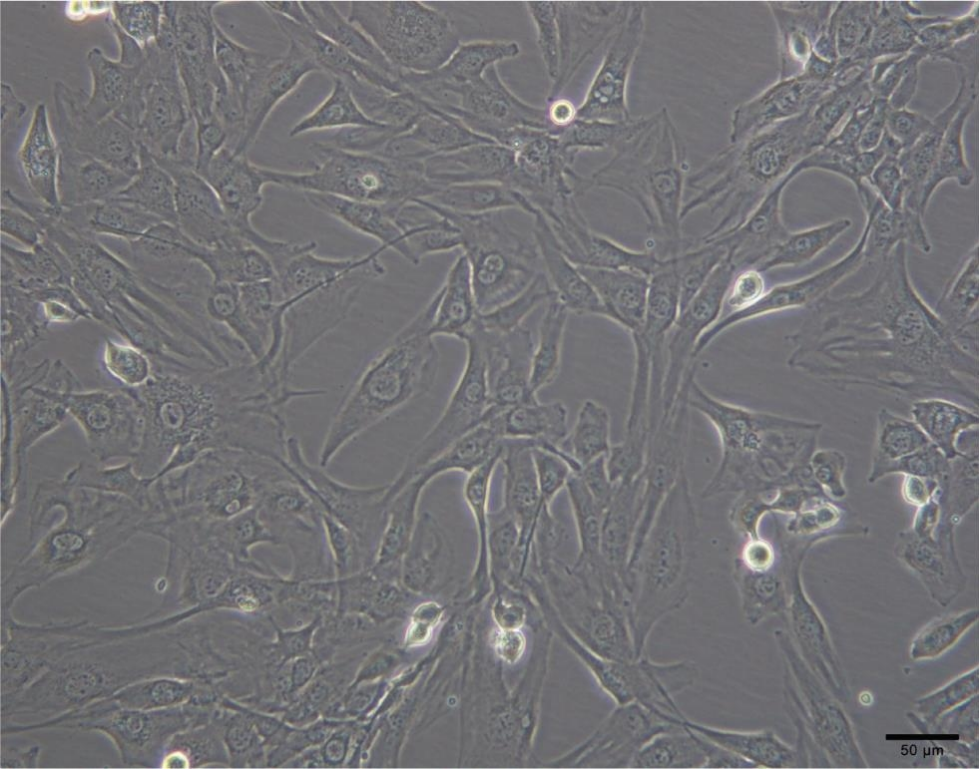
(C)

**Figure:**
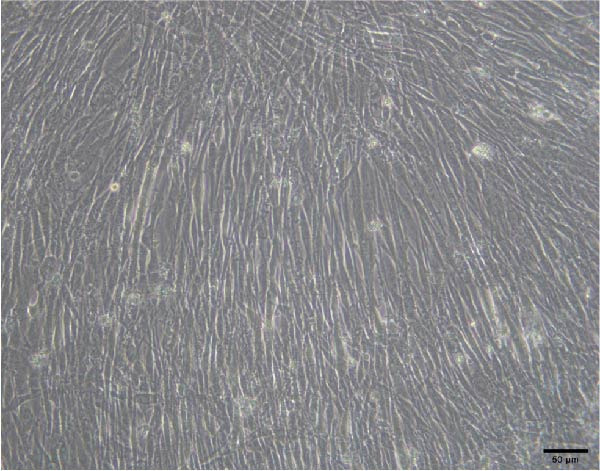
(D)

Figure 4Morphology of myotubes in each group under a microscope at 200×. (A) Myotube morphology of the control group; (B) Myotube morphology of the TNF‐α group; (C) Myotube morphology of the BMSC‐Exos group; (D) Myotube morphology of the BMSC‐Exos (GW4869) group.

**Figure:**
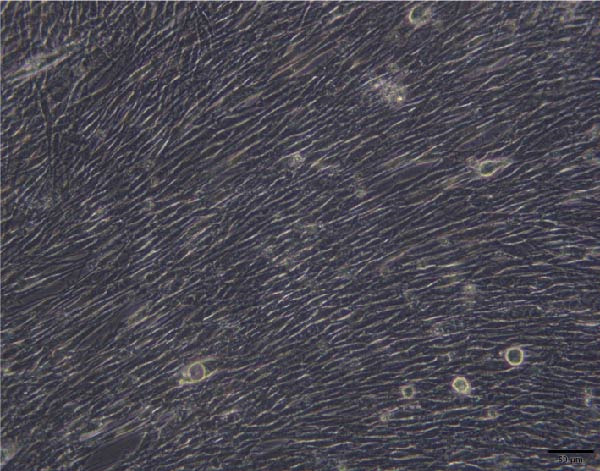
(A)

**Figure:**
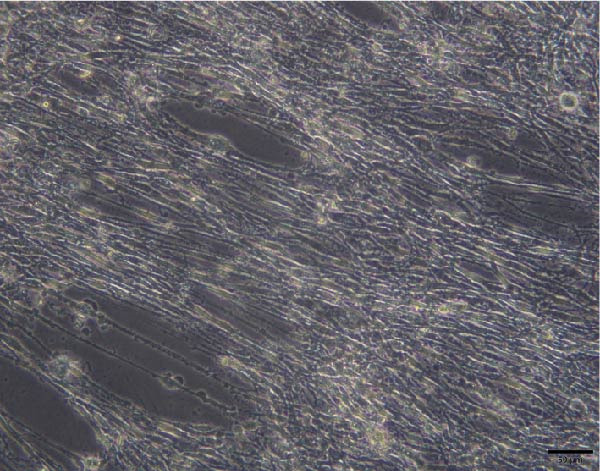
(B)

**Figure:**
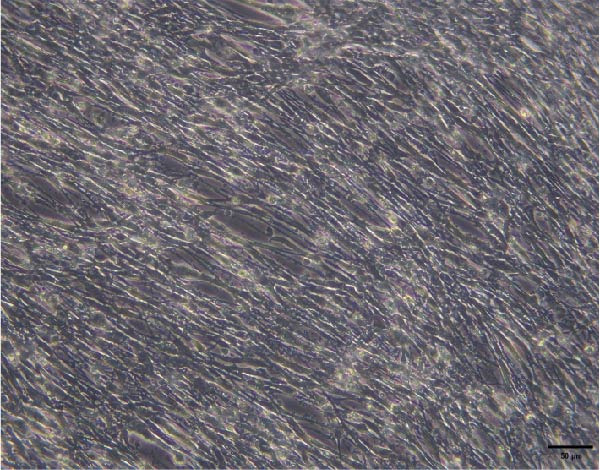
(C)

**Figure:**
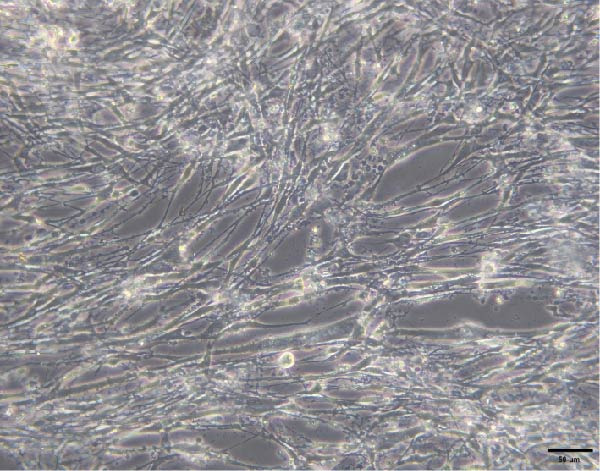
(D)

### 3.4. Uptake of Exosomes by Myotubes

**Figure 5:**
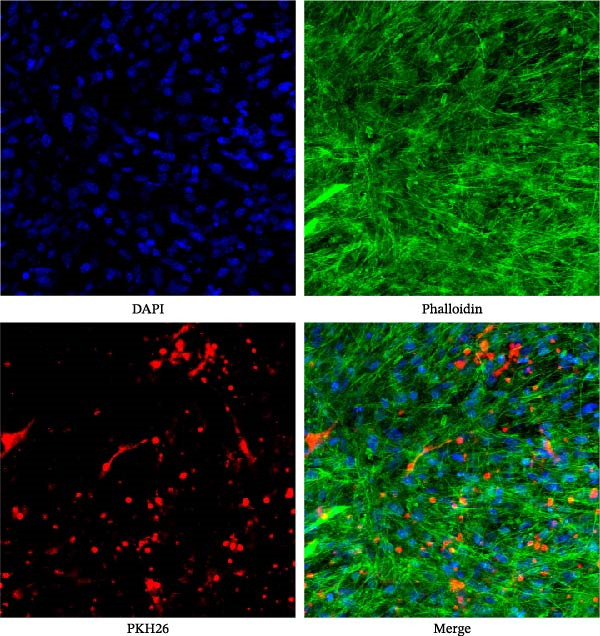
C2C12 myotubes were incubated with 100 μg/mL PKH26‐labeled exosomes for 48 h. After fixation and permeabilization, cells were stained with phalloidin (green, cytoskeleton) and DAPI (blue, nuclei). Red fluorescence indicates PKH26‐labeled exosomes, which were clearly internalized into the cytoplasm of myotubes and colocalized with the cytoskeleton. Scale bar = 50 μm.

To confirm the internalization of exosomes by C2C12 myotubes, confocal laser scanning microscopy was performed after incubation with PKH26‐labeled exosomes. As illustrated in Figure 5, robust red fluorescent signals (PKH26‐labeled exosomes) were clearly observed in the cytoplasm of C2C12 myotubes, and these signals were well colocalized with the green cytoskeleton stained by phalloidin, indicating that exosomes were effectively taken up by myotubes rather than merely adhering to the cell surface. DAPI staining (blue) clearly labeled the nuclei, further confirming the intracellular distribution of exosomes. Collectively, these results provide direct visual evidence that exosomes can be efficiently internalized into C2C12 myotubes, laying a foundation for their subsequent intracellular regulatory effects.

### 3.5. Effects of the BMSC‐Exos on Cell Viability

**Figure 6:**
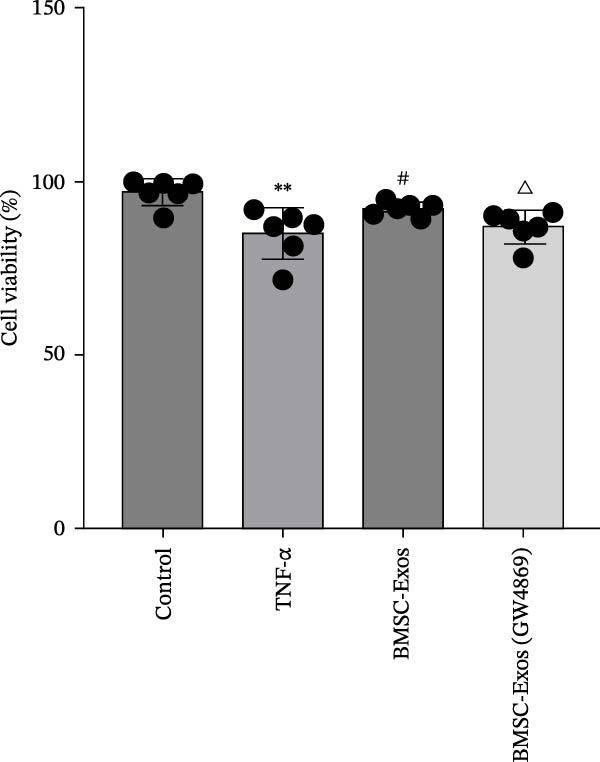
Effects of TNF‐α and BMSC‐Exos on C2C12 cell viability. TNF‐α: the viability of C2C12 cells treated with 20 ng/mL TNF‐α for 48 h. BMSC‐Exos: the viability of C2C12 cells treated with 20 ng/mL TNF‐α and BMSC‐Exos for 48 h. BMSC‐Exos (GW4869): the viability of C2C12 cells treated with 20 ng/mL TNF‐α and BMSC‐Exos (isolated from BMSCs treated by 10 μmol GW4869) for 48 h.  ^∗^^∗^ *p* < 0.01 vs. control untreated cells; ^#^ *p* < 0.05 vs. TNF‐α‐treated cells; ^△^ *p* < 0.05 vs. BMSC‐Exos‐treated cells.

To examine the protective effects of BMSC‐Exos against TNF‑α‑induced myotube damage, mouse skeletal muscle C2C12 cells were incubated with BMSC‐Exos for 48 h, and cell viability was measured via a CCK‐8 assay. The viability of TNF‐α‐treated myotubes was lower than that of nontreated myotubes. However, treatment with BMSC‐Exos protected cells from the TNF‑α‑induced, and the cell activity of the BMSC‐Exos (GW4869) group was slightly lower than that of the BMSC‐Exos group (Figure 6).

### 3.6. Effects of BMSC‐Exos on Myotube Diameter

**Figure 7:**
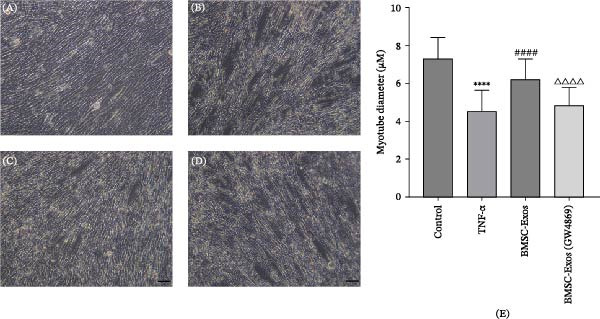
Effects of TNF‐α and BMSC‐Exos on C2C12 cell diameter. (A–D) Representative images of myotubes in each group: (A) Control group, (B) TNF‐α group, (C) BMSC‐Exos group, (D) BMSC‐Exos (GW4869) group. (E) Quantification of myotube diameters for C2C12 myotubes treated with 20 ng/mL TNF‐α and BMSC‐Exos for 48 h. Images were captured at 200× magnification (scale bar, 50 μm). The data are presented as the means ± standard deviations of three independent experiments.  ^∗∗∗∗^ *p* < 0.0001 vs. untreated cells; ^####^ *p* < 0.0001 vs. TNF‐α‐treated cells; ^△△△△^ *p* < 0.0001 vs. BMSC‐Exos‐treated cells.

As shown in Figure 7, the myotube diameter was measured. Compared with that of non‑treated myotubes, the diameter of TNF‑α‑treated myotubes was decreased. However, TNF‑α‑treated myotubes presented a significant increase in myotube diameter in the treatment with BMSC‐Exos; the cell diameter of the BMSC‐Exos (GW4869) group was slightly smaller compared to the BMSC‐Exos group (Figure 7).

### 3.7. Effects of BMSC‐Exos on the Ubiquitin‐Proteasome System

**Figure 8:**
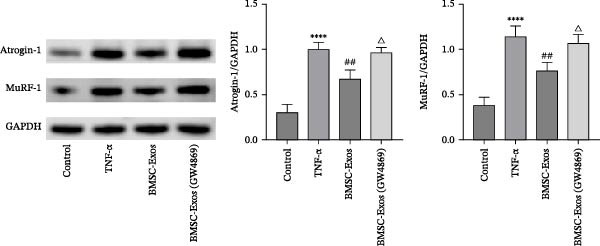
Effects of BMSC‐Exos on the ubiquitin‑proteasome system in TNF‑α‑treated C2C12 myotubes. Atrogin‐1 and MuRF‐1 protein expression levels were detected via western blot analysis. GAPDH was used as an internal standard. The data are presented as the means ± standard deviations of three independent experiments . ^∗∗∗∗^ *p* < 0.0001 vs. untreated cells; ^##^ *p* < 0.01 vs. TNF‐α‐treated cells; ^△^ *p* < 0.05 vs. BMSC‐Exos‐treated cells.

To confirm that the ubiquitin‐proteasome system is regulated by NF‑κB, the protein expression levels of E3 ubiquitin ligases in C2C12 myotubes were investigated via Western blot analysis. As shown in Figure 8, TNF‑α treatment significantly increased Atrogin‐1 and MuRF‐1 expression levels. However, the TNF‑α‑mediated upregulation of Atrogin‐1 and MuRF‐1 was reversed by treatment with BMSC‐Exos, and the expression levels of Atrogin‐1 and MuRF‐1 in the BMSC‐Exos (GW4869) group were higher than those in the BMSC‐Exos group (Figure 8).

### 3.8. Effects of BMSC‐Exos on Myogenesis

**Figure 9:**
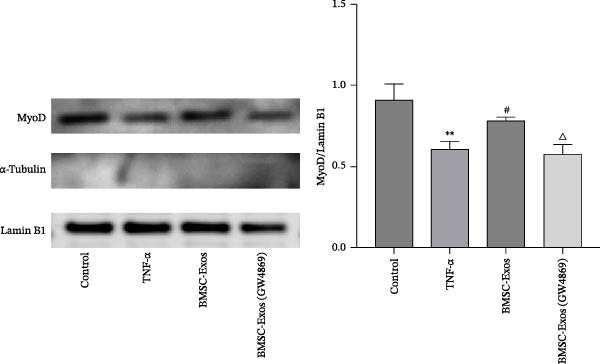
Effects of BMSC‐Exos on the expression of MyoD in TNF‑α‑treated C2C12 myotubes. Western blot analysis was performed to detect the protein expression levels of MyoD in nuclear fractions of C2C12 myotubes. α‐Tubulin (cytosolic marker) was undetectable in the nuclear fractions, confirming the high purity of the isolated nuclear components, while Lamin B1 was used as the nuclear loading control. The relative expression of MyoD was normalized to Lamin B1. The data are presented as the means ± standard deviations of three independent experiments.  ^∗∗^ *p* < 0.01 vs. untreated cells; ^#^ *p* < 0.05 vs. TNF‐α‐treated cells; ^△^ *p* < 0.05 vs. BMSC‐Exos‐treated cells.

To determine whether BMSC‐Exos treatment altered myogenic differentiation in TNF‑α‑treated C2C12 myotubes, MyoD protein expression levels were examined via Western blot analysis. As shown in Figure 9, MyoD protein expression levels were significantly lower in TNF‑α‑stimulated C2C12 myotubes than in control untreated myotubes. However, the TNF‑α‑mediated downregulation of MyoD was reversed by treatment with BMSC‐Exos; the MyoD expression level in the BMSC‐Exos (GW4869) group was slightly lower than that in the BMSC‐Exos group (Figure 9).

### 3.9. Effects of BMSC‐Exos on the NF‑κB Signaling Pathway

**Figure 10:**
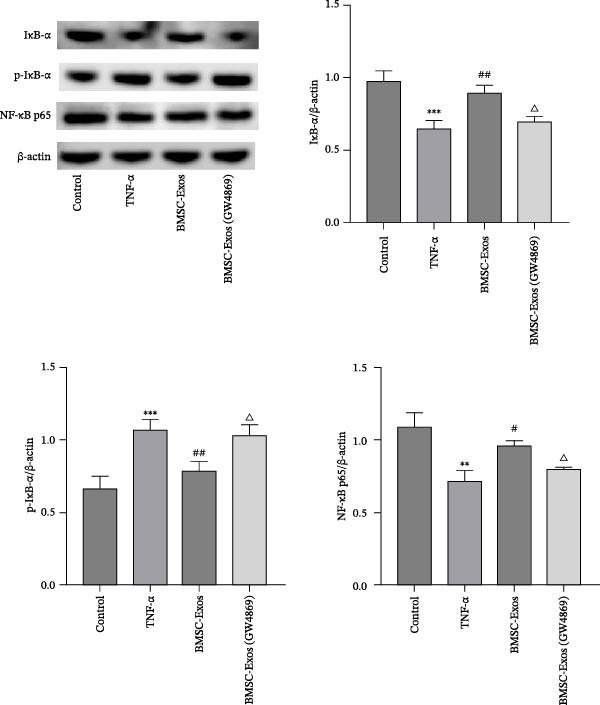
Effects of BMSC‐Exos on the activation and translocation of NF‑κB/p65 in TNF‑α‑treated C2C12 myotubes. IκB‐α, p‐IκB‐α, and NF‑κB/p65 protein expression levels in the cytosolic fractions were detected via Western blot analysis, and β‐actin was used as an internal standard. The data are presented as the means ± standard deviations of three independent experiments.  ^∗∗∗^ *p*  < 0.001,  ^∗∗^ *p*  < 0.01, vs. untreated cells; ^##^ *p*  < 0.01, ^#^ *p*  < 0.05, vs. TNF‐α treated cells; ^△^ *p* < 0.05 vs. BMSC‐Exos‐treated cells.

**Figure 11:**
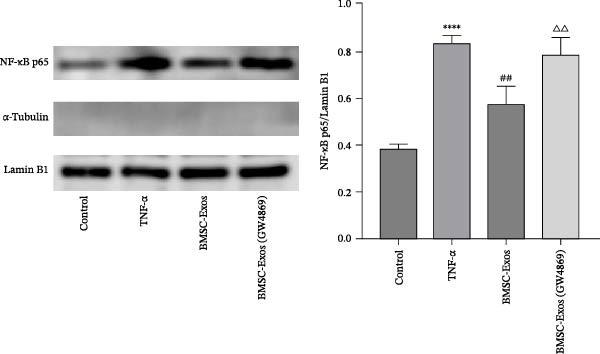
Western blot analysis was performed to detect the protein expression levels of NF‐κB p65 in nuclear fractions of C2C12 myotubes. α‐Tubulin (cytosolic marker) was undetectable in the nuclear fractions, confirming the high purity of the isolated nuclear components, while Lamin B1 was used as the nuclear loading control. The relative expression of NF‐κB p65 was normalized to Lamin B1. The data are presented as the means ± standard deviations of three independent experiments.  ^∗∗∗^ *p*  < 0.0001 vs. untreated cells; ^##^ *p*  < 0.01 vs. TNF‐α treated cells; ^△△^ *p* < 0.01 vs. BMSC‐Exos‐treated cells.

To further explore the mechanism of myotubes treated by BMSC‐Exos, myotubes were treated with TNF‑α (20 ng/mL) and BMSC‐Exos for 48 h, and proteins associated with NF‑κB/p65 subunit nuclear translocation were examined via western blot analysis. As shown in Figure 10, the phosphorylation of IκBα, which induces NF‑κB/p65 activation, was inhibited by BMSC‐Exos treatment in TNF‑α‑induced myotubes (Figure 10). Consequently, the nuclear NF‑κB/p65 levels were significantly lower in BMSC‐Exos‐treated C2C12 myotubes than in TNF‑α‐treated C2C12 myotubes (Figure 11). The results indicated that BMSC‐Exos treatment effectively blocked the nuclear translocation and activation of NF‑κB/p65 by inhibiting TNF‑α‑induced IκB‐α phosphorylation.

## 4. Discussion

MSCs are characterized by their self‐renewal and multiple differentiation potential, which allows them to regenerate various tissues and organs. EVs act as intercellular communication carriers containing bioactive substances, including cytokines, proteins, lipids, mRNAs, miRNAs, noncoding RNAs, and ribosomal RNAs. Their external lipid bilayer protects them from degradation by ribonucleases and enhances their biological efficiency in recipient cells. When secreted from host cells into recipient cells, the EV membrane directly fuses with the recipient cell membrane. Compared to traditional gene therapy carriers, EVs act as natural nanocarriers to protect and transfer biological factors, including miRNA, siRNA, or Antago miR, to recipient cells. Multiple studies have shown that EVs may be more effective than MSCs in stem cell‐based therapies. They can replace traditional BMSC therapy by transporting contents to target cells through membrane fusion or endocytosis. This provides cell‐free therapies such as repairing tissue damage, inhibiting inflammation, and regulating the immune microenvironment, thus offering new possibilities for targeted therapy.

Skeletal muscle atrophy is a common sign of aging. However, it can also result from disuse and various pathological conditions. During the aging process, the protein synthesis rate of skeletal muscles declines, while the degradation rate increases. This imbalance leads to muscle fiber atrophy, decreased muscle strength, and diminished physiological function of the muscles [28]. Recent advancements in understanding the mechanisms and related signaling pathways controlling skeletal muscle protein metabolism have significantly progressed in muscle atrophy research.

C2C12 myoblasts are commonly used in vitro to examine skeletal muscle atrophy. In this study, a 20 ng/mL TNF‐α model of skeletal muscle atrophy was established [29]. The results revealed that BMSC‐Exos protected C2C12 myotubes from TNF‐α‐induced damage, improved cell viability, and alleviated myotube atrophy. BMSC‐Exos inhibited protein degradation by suppressing Atrogin‐1 and MuRF‐1 expression and upregulated MyoD, indicating that they exert anti‐atrophic effects by modulating both protein synthesis and degradation. Confocal microscopy confirmed that PKH26‐labeled BMSC‐Exos were effectively internalized into C2C12 myotubes, providing direct evidence for their intracellular mechanism of action. Western blotting showed that BMSC‐Exos inhibited TNF‐α‐induced myotube atrophy by regulating the NF‐κB signaling pathway, and the neutral sphingomyelinase inhibitor GW4869 blocked exosome release from BMSCs and abrogated their protective effects. Overall, BMSC‐Exos exert antimyotube atrophy effects by improving cell viability, balancing protein metabolism, and inhibiting the NF‐κB pathway, with efficient internalization into myotubes as the critical cellular basis.

In summary, this study confirms that BMSC‐Exos mitigate TNF‐α‐induced C2C12 myotube atrophy by inhibiting the NF‐κB signaling pathway, which is reflected in restored myotube viability and diameter, downregulated Atrogin‐1/MuRF‐1, and upregulated MyoD. These in vitro findings establish a mechanistic basis for exploring BMSC‐Exos as a candidate for muscle atrophy intervention. In previous studies, besides the TNF‐α‐induced myotube atrophy model, other models of muscle atrophy have been established, including those induced by dexamethasone, starvation, rapamycin, the chemotherapeutic drug cisplatin, lipopolysaccharide (LPS), cancer cell‐induced atrophy, and denervation‐induced muscle atrophy [30–36]. Our study employed a single TNF‐α‐induced myotube atrophy model based on C2C12 cells, which is an immortalized myoblast cell line. To further enhance the translational value and universality of our findings, we will also validate the protective effect of BMSC‐Exos in primary myocytes and human‐derived muscle cells in future research, in addition to further investigating the role of exosomes in muscle atrophy models induced by other conditions, as well as whether they exert their effects through the NF‐κB signaling pathway or other alternative signaling pathways.

miRNAs and related proteins play crucial roles in the occurrence and development of muscle atrophy. For instance, miR‐486 is essential for normal muscle function and serves as a driver of myopathological remodeling in muscular dystrophy; overexpression of miR‐486 may be an effective approach to restore key dysregulated signaling pathways that affect growth and muscle function in dystrophic muscles [37]. miR‐26a overexpression in muscle can prevent muscle atrophy induced by chronic kidney disease [38]. In a dexamethasone‐induced muscle atrophy model, BMSC‐Exos inhibit dexamethasone‐induced muscle atrophy through the miR‐486‐5p/FoxO1 axis, and miR‐486‐5p inhibitors reverse the protective effect of BMSC‐Exos [39]. Overexpression of HSP70 inhibits disuse‐induced FOXO transactivation and prevents myofiber atrophy [40], while excessive expression of mitochondrial HSP60 promotes mitochondrial dysfunction and muscular dystrophy [41]. In the present study, whether BMSC‐Exos exert their effects by regulating the expression of miRNAs and related HSP proteins remains to be further verified, and we will continue our efforts to confirm this in future research.

This study has two notable limitations. First, it relies exclusively on the C2C12 myotube in vitro model, with no in vivo animal experiments conducted. The in vitro setting cannot fully replicate the complex in vivo microenvironment (e.g., systemic immune interactions and interorgan crosstalk), which restricts the direct clinical translational value of our conclusions. Second, we did not assess functional indicators of myotubes—such as contractile capacity or muscle fiber type switching—key metrics that reflect the core functional decline of skeletal muscle atrophy, as morphological and molecular changes do not fully align with functional outcomes.

In future research, we will address these limitations by performing in vivo animal experiments to validate the protective effects of BMSC‐Exos in mouse models of muscle atrophy and by conducting comprehensive functional assessments of muscle contractile activity. These efforts will help bridge the translational gap between our current in vitro mechanistic findings and potential clinical applications and will further verify the therapeutic potential of BMSC‐Exos for the treatment of muscle atrophy. In addition, we will explore the specific exosomal miRNAs and downstream molecular targets that mediate the anti‐atrophic effects of BMSC‐Exos, which will deepen our understanding of the underlying mechanisms.

## Author Contributions

Yibing Ke and Yongping Wang conceived and designed the study. Yibing Ke prepared the draft of the manuscript, including the figures and table.

## Acknowledgments

The authors have nothing to report.

## Funding

This work was supported by the Academic Enhancement Support Program of Hainan Medical University (Grant XSTS2026092).

## Disclosure

All the authors read and approved the final manuscript.

## Ethics Statement

This study was approved by the Ethics Committee of Lanzhou University First Hospital (LDYYLL‐2024‐426).

## Consent

The authors have nothing to report.

## Conflicts of Interest

The authors declare no conflicts of interest.

## Data Availability

The datasets used and/or analyzed during the current study are available from the corresponding author upon reasonable request.
